# The New Globalisation: From Too Big to Fail to Too Intertwined to Decouple

**DOI:** 10.1007/s10272-022-1089-7

**Published:** 2022-12-16

**Authors:** Simone Urbani Grecchi

**Affiliations:** International Strategic Analysis, Intesa Sanpaolo, Via Monte di Pietà, 8, 20121 Milan, Italy

**Keywords:** F23, F52, F53, H77

## Abstract

Going forward, international elites should instead strive for the application of one of the most liberal-minded principles of this current world order: self-determination.

Deglobalisation is a multifaceted phenomenon that has been going on for quite some time now, both at the private and public level. Under the pressure of an ever-changing geopolitical context, over the last two decades businesses and countries alike have elected to review their international positioning. But while the decision to relocate offshore activities for the corporate world hinges primarily (if not exclusively) on economic reasons, nation states work on different premises. Specifically, countries need to address (i) the widespread discontent and exacerbated inequalities caused by globalisation, which has failed to benefit the wider population; (ii) a growing sense of insecurity driven by conflicts, terrorism, cyber threats and unstable energy supplies; and (iii) their electorate's legitimate desire to see some jobs repatriated.

Clearly, there are economic, as well as financial, political and social reasons behind these deglobalising processes. But there is one underlying theme that brings all these aspects together: a heightened risk perception. Indeed, deglobalisation is a direct consequence of an increased level of mutual mistrust that characterises today's geopolitical ties around the world, which is perhaps the single most important failure of globalisation. Despite having brought together all the world's powers in multiple international fora, globalisation has so far failed to improve their reciprocal understanding. This is a costly failure (Jacques, 2022; and McCrea, 2020) because businesses will not spontaneously incur the cost of re/near/friend-shoring if risks abroad are perceived as manageable. Similarly, governments will not spend billions in subsidies, tax breaks and incentives to specific sectors if they believe that dependency on the skills or resources of a specific country did not pose a threat.

* The views expressed in this paper are entirely the author's and may not represent those of his employer.

Interestingly, however, and perhaps in a departure from other deglobalisation periods of the past (Kornprobst and Wallace, 2021), what characterises today's deglobalisation processes is that the world appears to be far too connected to become significantly deglobalised, making any attempt at a large-scale retrenchment rather limited in scope. In sum, in today's world, there is only a certain level of self-reliance that can be achieved.

To be sure, mutual misunderstanding does not seem set to abate any time soon. However, there are multiple counterbalancing effects that will likely keep deglobalisation at bay. Indeed, in spite of all the trade wars, even the US and China appear to be inextricably connected in terms of (i) trade, with goods and services exchanged between the two countries reaching US $550 billion in 2020; (ii) in the science field, where the two nations show a very high level of bilateral cooperation (Lee and Haupt, 2022); and (iii) financially – with China being the second major holder of US Treasuries (behind Japan) with US $971 billion (US Department of the Treasury, 2022). All of the aforementioned aspects are a double-edged sword, which is likely going to keep Washington and Beijing inextricably tied, perhaps even beyond their current political will.

However confrontational these two blocs may be, it is likely that they will depend on each other for a protracted period of time – if not for production, most certainly for mutual trade purposes, making cooperation, however begrudging, almost a necessity. Besides, should decoupling be extended to commercial exchanges, both Washington and Beijing would still need to find alternative markets for their goods and services. A tall order, given the sheer size of the American and Chinese consumer markets with respect to those of other countries. Furthermore, decoupling would be painful even from a capital markets standpoint. There has been much talk lately about delisting several Chinese companies from the US markets. Two things spring to mind. First, the listing of Chinese companies in the US has been going on unfettered both under the Obama and Trump administrations. As a result, today there are 264 Chinese/Hong Kong-based companies listed in the three main US bourses, for a market capitalisation as of September 2022, of around US $775 billion (US-China Economic and Security Review Commission, 2022). Second, even though the geopolitical pressure is mounting on both sides, western investors have not fled China (Nikoladze,

2022) in spite of its opacity, strict regulations on withdrawal and repatriation of capital and earnings (Newman et al., 2022), with more than one million foreign companies registered in China as of November 2021 (Registration China, 2022). All in all, while bilateral relations between the US and China are going to be tense and a cause of concern for the international order for the foreseeable future, such ties are so inextricable that they could actually slow deglobalisation down.

The war in Ukraine is reshaping today's international order. However, it is worth noting that only 19% of the international community (representing 59% of the world's GDP) has actually imposed sanctions on Moscow. Hence, expectations that the current war might be a game changer – specifically for the Western camp – are probably overblown. Two recent examples spring to mind. The EU has increased its LNG imports from Russia since February (European Commission, 2022), while Japan has recently pledged its commitment to Moscow for the Sakhalin-1 gas project. And even the announced NATO expansion to include Sweden and Finland may still not deliver a change of paradigm. In all likelihood, it should lead to a more effective military deterrence, with stronger naval capabilities in the Baltic region. This notwithstanding, priorities (China and the Indo-Pacific for the US; the fight against terrorism for Western Europe and Russia's threats for Eastern Europe) will likely continue to differ among NATO member states. Given that the alliance is unlikely to abandon its unanimity-based voting any time soon, it remains to be seen if NATO's effectiveness will actually improve after the announced enlargement.

Rather, what appears to be increasingly clear is that hostilities in Ukraine may significantly redefine relations across Asia. Firstly, between Central Asian nations and Moscow, with Kazakhstan taking a neutral stance over the conflict in Ukraine, Kyrgyzstan cancelling the annual joint military drills with Russia and Uzbekistan rekindling its quest for compensation over the Stalinist crimes under the USSR regime. Secondly, China and Russia will for the foreseeable future enjoy closer ties than in the past. Nevertheless, in the long run, such ties may look like a "marriage of convenience" more than a deeply rooted alignment. Moscow and Beijing are indeed on two different trajectories and could find themselves at odds over strategic dossiers like the Arctic – or even the access to Siberia's natural resources. Consequently, should the Ukraine war weaken Moscow, Novosibirsk could become more independent and develop closer ties with Beijing (fresh off the slogan "security and self-reliance", coined at the recent Chinese Communist Party congress), much to the detriment of the Russian federal central authorities and their control over Siberia.

A more multipolar world is therefore a likely scenario. However, those poles will not be set in stone, as we are moving towards a liquid international order, one in which relations will be variable and adaptable to specific circumstances – an era of "variable geometry". The seeds for this new, fractious environment have long been planted and have never ceased to germinate. Indeed, more than following a course of action defined at the multilateral/supranational level, countries have always tended to behave in very opportunistic ways by changing their foreign policy over a short span – even as short as a parliamentary term – and doing whatever suits them at a specific juncture. The same US-China relation is proof of that, with Washington periodically raising the human rights red flag on China or Saudi Arabia, while maintaining strong business ties with both. Another case in point is that of Turkey and Greece, who, irrespective of their NATO membership, never stopped purchasing military equipment from Russia (Petrov, 2007). Or even India, at the same time abstaining from voting against Russia at the United Nations over the war in Ukraine – all the while being part of the US-led Quadrilateral Security Dialogue (alongside Australia and Japan; Nazca, 2022).

Critically, this new setting will benefit neither multilateral organisations nor nation states. Indeed, the multilaterals will be lacerated from within by the growing competition among great powers. Furthermore, key players like Beijing are going to push for additional power. And precisely because China's role in today's international order is already tangible (e.g. in the United Nations Security Council), such strategy will be countered by the US and the West in general, generating a struggle that is going to render international organisations even more ineffective than they have been so far.

Indeed nowadays, more often than not, international bodies seem to be held hostage by their member states, who seem to use the multilateral platform to push their national agenda rather than pursuing the global common good. Against this backdrop, then, an argument could be made that the world has hardly ever experienced a fully fledged multilateralisation. Take the World Trade Organization (WTO). Considered by many to be the posterchild of the post-World War II, Western-led world order, it has been only partially effective, having allowed the signing of over five hundred regional trade agreements. While extremely beneficial for their signatories, these agreements de facto disregard one of the organisation's founding principles, i.e. the most-favoured-nation clause, designed to extend any favourable trade clause foreseen by a bilateral trade agreement to all WTO members. Another case in point is the World Health Organization, an organisation which can freely use only 16% of its funds (assessed contributions, made primarily by members states) – with the rest (voluntary contributions) being tightly allocated to projects specified by donors, the biggest of them privately owned companies (World Health Organization, 2022).

And if international organisations find it increasingly difficult to manage the global commons, nation states and their Westphalian model are going to appear even more ill-suited for the job. Central governments have lost control (at least partially) over security (both physical and cyber), financial flows, energy supplies and information. Critically, in many cases they seem to have lost control over the aspirations of local communities to self-determination as well, a founding principle of the UN charter and yet often overlooked – and, because of that, a source of social instability. Today, local as well as global factors are pulling at the fabric of the nation state, its stakeholders being at once local, cross-border and international, as is the case with ethnicities, debt bondholders or corporate shareholders.

Hence, a world order divided into two blocs, one under US and another under Chinese influence may not be a realistic scenario. China has a multitude of business partners, but very few allies. Besides, it remains to be seen whether Beijing's endgame is actually to form a bloc with like-minded nations, a case in point being perhaps Vietnam, which more often than not, finds itself at odds with its powerful neighbour. As per the US-led bloc, traditional values are going to hold, but so will national interests. Indeed, after the Ukraine war ends, the West will find it even harder to give globally (or simply regionally) coordinated responses, as natural gas is going to remain a viable source of energy for several countries in Central and Eastern Europe and East Asia, and gas pipelines connecting Russia to Europe may well end up resuming operations once hostilities in Ukraine terminate.

Just as importantly, one should caution against likening today's competition between the US and China to the US-USSR Cold War. Several aspects point to this conclusion. Firstly, the US and the USSR were two blocs squaring off militarily, while today there is hardly any evidence of a Chinese bloc. Secondly, the USSR was strong militarily but weak economically and financially, while China is a powerhouse in all these respects. Thirdly, unlike Washington and Beijing today, America and the USSR were not intertwined, with bilateral exchange limited to approximately 1% (Becker, 1987).

In what looks set to become an even more chaotic international arena, the ability to coexist will be key. This translates into moving beyond the simple dichotomy of "friend and foe" (Leonard, 2022) and sharpening whatever proclivity the world's countries have to listen to their peers' views. Besides, any measure reducing international exchanges of goods and services would have a negative impact on local economies and could prove to be a hard sell even for those political movements that, in principle, advocate autocratic models but, in reality, are deeply rooted in constituencies which are, in many advanced economies, significantly export-oriented. A glaring example of how important co-existence, openness and the ability to listen are going to be is the Ukraine war. The West had been aware of Russia's belligerent stance over Ukraine at least since 2007, which makes its lack of attention to the issue particularly questionable. Most certainly, as discussed by prominent analysts, it would be outrageous to blame the war on the West and NATO. But it should have been clearer to the West that by having Ukraine join the EU (something Moscow has never been opposed to; Daily Sabah, 2022), Kyiv could have been better protected from foreign attacks under the provisions of the Treaty on the European Union, e.g. Art. 42.7. Similarly, self-assigned quests to make the world a better place have led some countries in the West to misguided approaches such as, for instance, the notion that democracy can be exported (Iraq, Afghanistan) or the expectation that a given country's values are necessarily bound to become more liberal as it becomes richer (China).

Going forward, international elites should instead strive for the application of one of the most liberal-minded principles of this current world order: self-determination. A guiding principle which helped shape the history of mankind, self-determination is hardly at the forefront of today's international policymaking. Examples abound: Taiwan, Kurdistan, Bosnia, Kosovo. Of course, all these cases come with their own specificities. But for all of them, the underlying theme is to give a specific community willing to "go it alone" the opportunity to do so, underscoring the inalienable right of like-minded communities to cater for themselves locally and to join the international community on their terms. Sure, to "go small" comes with consequences. However, it could be argued that prosperous (albeit small) nations could be the cornerstone of a more peaceful international community.

In this context, the multilateral fora's reduced effectiveness may provide an opportunity for the European Union to assert itself. But will its member states keep it in the current limbo? Or will they eventually change track and allow the bloc to become a "geopolitical Europe"? Today, the EU remains an unfinished project, with its own member states still unwilling to assign to the supranational entity all the powers it needs to interact with Washington and Beijing on the same footing. Interestingly, only two years ago it was argued by some authoritative observers that the EU is a political powerhouse as well as a financial dwarf. Provided this has ever been the case, the situation seems to have somewhat flipped. Indeed, Brussels looks unable to punch to its (potential) political weight in the international arena. However, it has managed to raise its financial profile in the recent public healthcare and energy crises. Looking ahead, a newfound financial strength could signal that Brussels is perhaps inching closer to its political *Zeitenwende*. Should this be the case, on the verge of turning 31, the European Union may finally leave its parental home (that is, the veto power of its member states) and start a real life of its own.
